# Role of NEK2A in Human Cancer and Its Therapeutic Potentials

**DOI:** 10.1155/2015/862461

**Published:** 2015-02-01

**Authors:** Jiliang Xia, Reinaldo Franqui Machin, Zhimin Gu, Fenghuang Zhan

**Affiliations:** ^1^Cancer Research Institute, Southern Medical University, Guangzhou, Guangdong 510515, China; ^2^Department of Internal Medicine, University of Iowa, Carver College of Medicine, Iowa City, IA 52242, USA

## Abstract

Chromosome instability (CIN) has been identified as a common feature of most human cancers. A number of centrosomal kinases are thought to cause CIN in cancer cells. Part of those centrosomal kinases exhibit elevated expression in a wide variety of tumours and cancer cell lines. Additionally, critical roles in many aspects of cancer cell growth, proliferation, metastasis, and drug resistance have been assigned to some of these centrosomal kinases, such as polo-like kinase 1 (PLk1) and Aurora-A kinase. Recent studies from our group and others revealed that a centrosomal kinase, Never in Mitosis (NIMA) Related Kinase 2A (NEK2A), is frequently upregulated in multiple types of human cancers. Uncontrolled activity of NEK2A activates several oncogenic pathways and ABC transporters, thereby leading to CIN, cancer cell proliferation, metastasis, and enhanced drug resistance. In this paper, we highlight recent findings on the aberrant expression and functional significance of NEK2A in human cancers and emphasize their significance for therapeutic potentials.

## 1. Introduction

Cancer cells tend to show some degree of genetic instability. It is now clear that high genetic change or instability plays a major role in cancer development [1]. Genetic instability can trigger tumorigenesis mainly through the activation of oncogenes and/or the inactivation of tumor suppressor genes. Chromosome instability (CIN), a phenotype characterized by a high rate of gain and/or loss of whole or large portions of chromosomes at each cell division, has been implicated in the initiation of genetic instability [2]. CIN generates a disparity in chromosome number (aneuploidy) and an enhanced rate of loss of heterozygosity, which is frequently seen in cancer cells [3–5]. Theodor Boveri observed abnormal chromosome quantities in cancer cells as early as a century ago [6]. However it was only in the recent years that CIN has been positively correlated with tumorigenesis, cancer progression, and therapeutic resistance [3–5].

Former studies have indicated that defects in cell division, telomere stability, and the DNA damage response all contribute to CIN in cancer [7]. Numerous cell division related proteins, which are highly expressed in multiple cancers, are involved in the initiation of CIN in cancer cells [8, 9]. Centrosomal kinases are important regulators of cell division. Uncontrolled activity of centrosomal kinases can lead to spindle abnormalities, centrosome fragmentation, premature centriole splitting, multiple nucleuses, supernumerary centrosomes, and chromosome segregation errors. All those abnormal phenotype are important risk factors for CIN, indicating that overexpression of centrosomal kinases might drive tumor progression by promoting CIN [10, 11]. Studies from our group and others have demonstrated that elevated Never in Mitosis (NIMA) Related Kinase 2A (NEK2A), a member of the NIMA-related serine/threonine kinase family and a core component of centrosome, results in CIN in cancer cells [12, 13]. Importantly, our previous studies indicated that high expression of NEK2A is associated with poor survival in various cancers [12]. In recent years, a larger number of studies focused on the roles of NEK2A in tumorigenesis, cancer progression, and drug resistance have been published. In view of previous studies, we speculated that NEK2A may be a novel potential biomarker for diagnosis and a possible therapeutic target for human cancers.

## 2. Basic Biology of NEK2A and Validated Functions of NEK2A in Normal Cells

**Figure 1:**
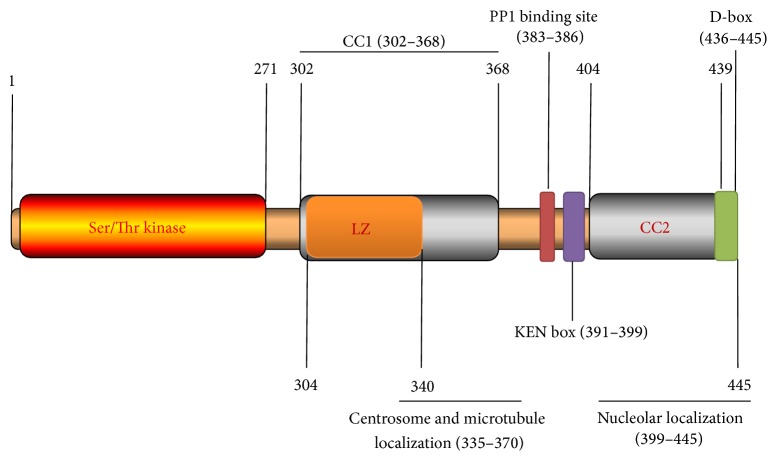
NEK2A protein structure. The relative positions of the catalytic domain (serine/threonine kinase), leucine zipper (LZ), coiled coil (CC), PP1 binding site, centrosome localization microtubule site, nucleolar localization, KEN-box, and D-box are indicated. Numbers above and below the structures indicate amino acid positions.

The NEK2 gene in humans is located in chromosome 1 and it is comprised of 8 exons. There are three isoforms that result from the alternate splicing of this gene, termed NEK2A, NEK2B, and NEK2C. NEK2A is the most studied isoform and it is a cell cycle-regulated kinase structurally related to the mitotic regulator NIMA of* Aspergillus nidulans*, being 47% identical within the catalytic domains [14]. NEK2A is also 31% structurally identical to Aurora-A, a human serine/threonine kinase involved in proper cell division [15]. Other structural studies demonstrated that human NEK2A is a 445 amino acid (48 kDa) protein comprising an N-terminal kinase domain and a C-terminal noncatalytic regulatory domain [15]. The NEK2A N-terminal kinase domain has all the motifs typical of a serine/threonine kinase. The C-terminal region possesses multiple regulatory motifs, which regulate the activity, location, and stability of NEK2A. These include leucine zipper (LZ), coiled coil (CC), centrosome, microtubule and nucleolar localization sites, PP1 binding site (KVHF), and APC binding site KEN-box and extended cyclin A-type destruction box (D-box) (Figure 1) [15].

Subcellular localization analysis shows that NEK2A resides in both the nucleus and cytoplasm throughout the cell cycle [16]. More detailed localization studies on the cytoplasmic NEK2A concurred to show that it is a core component of the centrosome [17]. In addition, NEK2A has been detected at nucleoli in interphase cells, on condensed chromatin in meiotic and mitotic cells, and at the kinetochores and midbody of dividing cells [16–20]. Western blot analysis demonstrates that NEK2A displays a cell cycle-dependent expression pattern, being low in G1, increasing through S and G2 to reach peak in late G2/M, and decreasing upon entry into mitosis [14, 21]. Several studies have shown that a key mechanism that maintains NEK2A suppressed during M phase is the ubiquitin proteasomal system (UPS) [22]. NEK2A degradation through the UPS depends on direct binding of NEK2A to the Anaphase Promoting Complex (APC/C) via two C-terminal motifs including the D-box and the KEN-box [22, 23]. This interaction leads to the ubiquitination of NEK2A and its degradation by the 26S proteasome. No protein, to our knowledge, has yet been identified to stabilize NEK2A through deubiquitination; however this could also represent another aspect of NEK2A regulation. Posttranslational modifications are not the only mechanism that keeps NEK2A regulated in a cell cycle-dependent manner. Negative transcriptional regulators, like E2F4, and the epigenetic modulators, p107 and p130, negatively affect NEK2A levels directly and indirectly, respectively [24].

Similar to its expression pattern, the activity of NEK2A is cell cycle-regulated, with maximum activity in S and G2 phases and low activity upon mitotic entry. NEK2A dimerization via the leucine zipper motif is essential for full activation, both in vitro and in vivo, most likely as a result of its promoting of transautophosphorylation [25]. This was shown by deleting the leucine zipper motif, which prevented the transautophosphorylation of NEK2A and reduced NEK2A activity. Many possible autophosphorylation sites of NEK2A were first identified by mass spectrometry in both the N-terminal catalytic domain and C-terminal regulatory domain [15]. Some of these have been confirmed with in vitro kinase assays and their physiological relevance with various cell lines. Of the most important autophosphorylation sites described thus far are T175 and T179, localized in the kinase domain, which allow activation of NEK2A [15]. Other autophosphorylation sites outside the kinase domain have been described, some in the KEN-box and others in the coiled coil region, suggesting a role in kinase regulation and dimerization, respectively [15]. More biochemical studies must be done to understand the role of these phospho-sites. NEK2A can be negatively regulated through dephosphorylation by Protein Phosphatase 1 (PP1) that directly binds to a KVHF sequence within the C-terminal of NEK2A protein [26, 27]. As expected, overexpression of PP1 suppresses NEK2A kinase activity, while depletion of PP1 by small interfering RNA showed increased NEK2A activity.

The subcellular localization, cell cycle-dependent expression, and activity together suggest that NEK2A may play an important role in cell division. Previous studies have demonstrated that some cell division related proteins interact with NEK2A (Table 1). Transfection of active, but not inactive NEK2A, exhibited a premature separation of centrosomes in the cell cycle, while depletion of NEK2A interferes with centrosome separation in G2 cells [17]. Subsequent studies further suggested that NEK2A induces centrosome separation by phosphorylating centrosome cohesion proteins C-Nap1 and Rootletin [28–30]. Besides centrosome separation, NEK2A also regulates microtubule organization through phosphorylation of ninein-like protein 2 (NLP2), resulting in its removal from the centrosome at the time of mitotic entry [31–35]. NEK2A can also help recruit numatrin to the centrosome through its kinase activity. Previously considered as noncentrosomal, recent data has surfaced that numatrin's recruitment to the centrosome protects against centrosome hyperamplification and genomic instability [36, 37].

Apart from its many functions in the centrosome, subcellular localization studies have found a fraction of endogenous NEK2A at condensed chromatin, particularly in cells undergoing meiosis. According to previous studies, NEK2A is activated by the MAPK pathway; it then phosphorylates an architectural chromatin protein, HMGA2. This phosphorylation decreases the affinity of HMGA2 for DNA and might drive its release from the chromatin, thereby promoting chromatin condensation [38, 39]. NEK2A has also been reported to regulate chromosome separation by modulating the spindle assembly checkpoint (SAC). NEK2A achieves this through direct interaction or phosphorylation of mitotic arrest deficient-like 2 (MAD2), mitotic arrest deficient-like 1 (MAD1), telomere repeat binding protein (TRF1), and highly expressed in cancer 1 (HEC1) [12, 40–42]. Some studies show that NEK2A regulates the alignment of chromosomes to the spindle (chromosome congression) through phosphorylating SGO1 at Ser14 and Ser507 [43]. Expression of nonphosphorylatable mutant SGO1 perturbed chromosome congression and resulted in a dramatic increase in microtubule attachment errors, including syntelic and monotelic attachments. In addition to participating in cell division, NEK2A was identified as a novel mRNA splicing factor kinase. NEK2A colocalizes in splicing speckles with SRSF1 and SRSF2, important splicing factors, and has been shown to phosphorylate the former [44]. Overexpression of NEK2A induces phosphorylation of endogenous SR proteins, a kind of proteins containing a protein domain with long repeats of serine and arginine amino acid residues, and affects the splicing activity of SRSF1 toward reporter minigenes and endogenous targets, independently of SRPK1. Conversely, knockdown of NEK2A, like that of SRSF1, induces expression of proapoptotic variants from SRSF1-target genes and sensitizes cells to apoptosis.

Although much progress has been made in our understanding of NEK2A in the past decades, several setbacks have slowed the progress in the study of this kinase. One of these is the lack of isoform-specific antibodies. Because of the similarity between NEK2A, NEK2B, and NEK2C, antibodies for each one are not available, making functional studies of endogenous NEK2A somewhat troublesome. Another problem involving the study of NEK2A is the toxicity of the wild type active protein in bacteria [66]. This does not allow the production of bulk NEK2A for crystallography experiments. The recent data uncovered by crystallography is based on unphosphorylatable mutants [15]. Another issue with NEK2A research is the lack of mouse models. The documented functional research of NEK2A is thus restricted to in vitro studies so far. To better characterize the in vivo role of NEK2A, mouse models of NEK2A are highly demanded. Our group has generated a NEK2A transgenic model, in which expression of NEK2A is turned on upon breeding with Cre mice in a tissue specific manner. As NEK2A is highly expressed in B cell lymphoma and multiple myeloma [12, 67], the NEK2A transgenic mice were bred with CD19 promoter driving cre to overexpress NEK2A in B cell lineage. Instead of developing B cell malignancies, these mice had altered B-cell development by increasing immature B-cells in the bone marrow and decreasing B-1 B-cells in peritoneal cavity. Furthermore, transgenic expression of NEK2A induced formation of spontaneous germinal centers and exhibits enhanced T-cell dependent immune response (unpublished data). All these provided the novel evidence of NEK2A's function in vivo. In addition, we are also developing NEK2A knockout mice using a gene trap strategy to better explore NEK2A's role in pathophysiological conditions.

## 3. Aberrant Expression of NEK2A in Human Cancers

Protein kinases that regulate the centrosome cycle are often aberrantly controlled in tumor cells. Changes in their expression can lead to CIN and aneuploidy, potentially triggering tumorigenesis. Increased expression of NEK2A has been reported in various cancer cells (Table 1). Reports implicating high expression of NEK2A in human cancer first appeared following microarray analysis of mRNA abundance in cancer cell line derived from Ewing tumors (ETs) (Table 2) [45]. Subsequent RT-PCR or Western blot analysis showed that multiple types of human cancer cell lines, including cholangiocarcinoma [46], testicular seminomas [47], human breast cancer [48–50], cervical cancer [50], prostate cancer [50], and colorectal Cancer [53, 54], expressed higher NEK2A in the level of mRNA or protein than normal human fibroblast cells. Consistently, analysis by Western blot, real-time PCR, DNA microarray, and immunohistochemistry indicated that increased NEK2A is found in various cancer tissues, such as human testicular seminomas [47], human breast carcinoma [49, 51, 52], colorectal cancer [53, 54], malignant peripheral nerve sheath tumors [55], nonsmall cell lung cancer [56], renal cell carcinoma [57], and pancreatic ductal adenocarcinoma [58]. Our previous gene expression profiling (GEP) analysis showed that NEK2A expression was significantly upregulated in several types of human cancer samples compared to normal cells, including multiple myeloma, myeloid leukemia, breast cancer, lung adenocarcinoma, mantle cell lymphoma, mesothelioma, head and neck squamous cell carcinoma, bladder carcinoma, glioblastoma, T-cell acute lymphoblastic leukemia, colon carcinoma, hepatocellular carcinoma, melanoma, and ovarian adenocarcinoma [12].

Though we know NEK2A is highly expressed in various cancer cells, the underling mechanisms of increased NEK2A in cancer cells still remain poorly understood. Since both mRNA and protein of NEK2A are increased in cancer cells, several tumor-associated transcription factors and posttranslational modifications may be involved in the high expression of NEK2A in cancer cells. MicroRNA-128, a tumor suppressor, is thought to target NEK2A in colorectal cancer cell [68]. Colorectal cancer patients with high miR-128 expression had significantly lower NEK2A expression and lower recurrence rates than those with low miR-128 expression. Consistent with other tumor suppressor microRNAs, microRNA-128 is silenced by DNA methylation in colorectal cancer cells. A two- to threefold recovery of miR-128 expression was found after 5-aza-2-deoxycytidine (5aza-dC) treatment, a DNA-demethylating agent. Moreover, NEK2A expression levels were significantly reduced after 5aza-dC treatment. In addition to being indirectly inhibited by demethylation, NEK2A transcript levels are reduced by direct demethylation in HCT116 colon cancer cells, which is restricted to the distal region of the NEK2A promoter, but not in isogenic p53^−/−^ cells [69]. Chromatin immunoprecipitation analysis demonstrated that p53 directly and specifically binds to the distal NEK2A promoter. Stabilization of endogenous p53 by doxorubicin or ectopic expression of p53, but not a p53 DNA-binding mutant, decreased NEK2A expression [69]. This study suggests that demethylation of the distal NEK2A promoter represses NEK2A expression in a p53-dependent manner. As mentioned previously, in G1 and M phase normal cells, NEK2A expression is downregulated by tumor suppressors including the retinoblastoma (Rb) family members p107 and p130 [24] and APC [22, 23]. Chromatin-immunoprecipitation (ChIP) assays demonstrated that the promoter of NEK2A is bound by E2F4 transcription factor in early G1 [24]. E2F4, a member of the E2F transcription factor family, interacts with Rb family members p107 and p130 and acts as a transcriptional repressor in G0 and G1 through recruitment of histone deacetylase which suppressed gene expression. In p107^−/−^ and p130^−/−^ mouse embryo fibroblasts (MEFs), the expression of NEK2A is significantly increased even in the absence of serum suggesting that tumours lacking p107 or p130 are likely to have elevated levels of NEK2A [24]. Moreover, overexpression of E7, a human papillomavirus encoded protein which represses the function of Rb family members, leads to increased NEK2A expression in human keratinocytes [70]. Forkhead transcription factor FOXM1 regulates the expression of many G2-specific genes including NEK2A and is essential for proper mitotic progression [71]. Overexpression of recombinant FOXM1 increases NEK2A expression; conversely, FOXM1 depletion reduces NEK2A expression. So far, very few reports about the relationship between NEK2A expression and tumor suppressors and oncoproteins in cancer cells have been published. Low expression of p130 and p107 or inactivated APC frequently occurs in the carcinogenic processes of multiple types of cancers [72, 73]. Both high expressions of FOXM1 and E7 are important risk factors for tumorigenesis [74, 75]. Thus elevated NEK2A in cancer cells may be induced by those abnormal conditions. Studies about the mechanisms of NEK2A expression regulation in cancers may contribute to clinical application of NEK2A-based anticancer therapeutics.

## 4. Roles of NEK2A in Tumorigenesis, Tumor Progression, and Drug Resistance

Former studies have demonstrated that NEK2A involves various signaling in a broad range of cancers (Table 3).

### 4.1. Tumorigenesis

As discussed above, studies have implicated NEK2A in the regulation of centrosome separation, microtubule organization, chromatin condensation, SAC, and chromosome congression during cell division. Overexpression of NEK2A in cancer cells may result in premature centriole splitting, spindle abnormalities, multinucleation, centrosome amplification (CA), and chromosome segregation errors. These cellular phenotypes ultimately lead to CIN and aneuploidy, which is frequently observed in transformed cells with overexpressed NEK2A. This suggests that overexpression of NEK2A triggers tumorigenesis by promoting CIN and aneuploidy. Consistent with this idea, our previous studies show that overexpression of NEK2A in multiple myeloma cell results in CIN [12].

Several cell division proteins and signaling pathways are involved in NEK2A mediated CIN and aneuploidy. The MAPK pathway is required for maintaining chromatin condensed during the two meiotic divisions and uncontrolled activity of MAPK pathway has been implicated in CIN [76]. Previous studies show that NEK2A is phosphorylated by the MAPK effector P90R^sk2^, thus placing these two proteins in the same pathway. Moreover, the induction of chromatin condensation requires the MAPK pathway and P90R^sk2^. Interestingly, inhibiting MAPK in the presence of okadaic acid prevents not only chromatin condensation, but also the activation of NEK2A [39]. So NEK2A may be involved in the MAPK induced CIN. The Hippo pathway components, MST2 and HSAV1, also have a direct interaction with NEK2A, thereby regulating its ability to localize to centrosome and phosphorylate C-Nap1 and Rootletin [59]. Polo-like kinase 1 (PLK1), a serine/threonine kinase identified as a potential drug target in cancer therapy, may also affect NEK2A activity in cancer cells, albeit indirectly. Tumors with PLK1 overexpression were associated more frequently with CIN (*P* < 0.0001), DNA aneuploidy (*P* = 0.0007), and CA (*P* = 0.0013) than those without PLK1 overexpression [77]. Functional studies have demonstrated that PLK1 can phosphorylate MST2, and this happens upstream of the MST2-NEK2A-induced centrosome separation [60]. The absence of PLK1 phosphorylation of MST2 promotes assembly of NEK2A-PP1*γ*-MST2 complexes, in which PP1*γ* counteracts NEK2A kinase activity. In contrast, PLK1 phosphorylation of MST2 prevents PP1*γ* binding to MST2-NEK2A, allowing NEK2A activity to promote centrosome separation. In addition to regulating MST2-NEK2A-induced centrosome separation, PLK1 was shown to promote the NEK2A-*β*-catenin-induced centrosome separation [61] and NEK2A-NIP-induced microtubule organization [32]. This suggests that PLK1 is an essential regulator of NEK2A in cancer cells. In summary, NEK2A has roles downstream of the MAPK pathway and PLK1; hence NEK2A may be involved in MAPK- and PLK1-induced CIN and tumorigenesis.

Abnormal expression of SAC proteins can cause cell aneuploidy, an important factor in tumorigenesis. High expressions of cell division cycle 20 homolog (CDC20) and MAD2, key components of SAC, have been reported in various carcinomas. Previous studies have demonstrated that NEK2A can phosphorylate MAD2 and CDC20. Moreover, overexpression of NEK2A acts upon the MAD2-CDC20 complex and induces a delay in mitosis, promoting aneuploidy in cancer [63]. HEC1, a Ndc80 complex protein localized at kinetochores and highly expressed in cancer, is phosphorylated by NEK2A at 165-serine [42]. Overexpression of HEC1 in an inducible mouse model results in mitotic checkpoint hyperactivation and is sufficient to generate tumors that harbor significant levels of aneuploidy in vivo [78]. Former studies have demonstrated that the phosphorylation of HEC1 by NEK2A is essential for MAD1 and MAD2 to localize to the kinetochores, which is involved in HEC1 induced tumorigenesis. Their studies suggest that HEC1, MAD2, and CDC20 may be involved in NEK2A induced CIN in cancer cells.

In Her2+ breast cancer cells, knockdown of NEK2A reduces CA and binucleation while its overexpression enhances CA [62]. Moreover, ectopic expression of NEK2A in immortalized HBL100 breast epithelial cells leads to accumulation of multinucleated cells with supernumerary centrosomes [50]. NEK2A expression is regulated by CDK4, which is a major regulator of CA in Her2+ breast cancer cells [62], suggesting that NEK2A may be a downstream target of CDK4, and is involved in CDK4 induced CA. Additionally, TRF1 was shown to be involved in NEK2A induced aneuploidy. It has been discovered that TRF1 interacts directly with and is phosphorylated by NEK2A. NEK2A overexpression in the breast cancer cell lines, MDA-MB-231 and MCF7, results in CA and multinucleation, which leads to aneuploidy; however TRF1 depletion by siRNA prevents this phenomenon [64]. Moreover, when exogenous TRF1 was added back in NEK2A-overexpressed cells with no endogenous TRF1, cells had reinduced cytokinetic failure.

As summarized above, the expression and activity of NEK2A are regulated by many tumor suppressors and oncoproteins that show aberrant behavior in cancer. This, coupled with the abundant evidence on the effects of NEK2A on cell physiology, strongly suggests that NEK2A is an oncoprotein capable of being deregulated by several pathways. On the other hand, NEK2A regulates the activity of some cancer-related proteins by interacting and phosphorylating them; hence NEK2A may be involved in the process of tumorigenesis.

### 4.2. Tumor Progression

Studies in multiple types of cancers have demonstrated that elevated NEK2A promotes cell proliferation, while its suppression with siRNA inhibited this proliferation and induced cell death [12, 46, 48–50]. Moreover, cancer cells overexpressing NEK2A showed a significant increase in colony formation compared with control cells [12, 48]. In a xenograft nude mouse model, subcutaneous injection of NEK2A siRNA around the tumor nodules resulted in reduction of tumor size compared with those of control siRNA injection [46, 48]. In a peritoneal dissemination model, NEK2A siRNA-treated mice showed statistically longer survival periods in comparison with those of the control siRNA treated mice [46]. Former studies show that NEK2A expression was positively associated with Ki-67 expression, a cell proliferation marker, in multiple myeloma, human primary breast cancer tissue, and nonsmall cell lung cancer [12, 49, 56]. In addition, NEK2A cytoplasmic expression was positively associated with cancer grade and tumor size in breast invasive ductal carcinoma (IDC) [51]. These data all point to NEK2A supporting tumor progression both in vitro and in vivo. Interestingly, Hayward et al. concluded that NEK2A upregulation appears to precede metastasis in their ductal carcinoma samples [50]. In line with this data, another group showed that colorectal cancer patients with high NEK2A mRNA showed greater lymph node metastasis, increased serosal, lymphatic, and venous invasion, and peritoneal dissemination when compared to the patients with low NEK2A mRNA [68]. Also, elevated NEK2A expression was maintained within all matched colorectal cancer metastases samples from NEK2A-overexpressing primary tumours. This suggests that overexpression of NEK2A may also precede metastasis and/or help the cells survive the process in this cancer. To shed some insight on the mechanisms of the metastasis-inducing potential of NEK2A, a study in Drosophila by the Paroly group demonstrated that dNek2 cooperates with Ras and Src signaling to promote metastasis. Coexpression of dNek2 along with activated Ras and Src (dNek2; Csk^−/−^; Ras^V12^ cell) led to significant overgrowth of tumor cells as well as appearance of secondary tumors in the body of the larvae. In tumor cell injection assays, dNek2; Csk^−/−^; Ras^V12^ tumor cells were injected into the dorsal notum region of wild type (WT) adult flies, and within 10 days of injection tumor cells could be seen in various parts of the adult body. However, injection of dNek2 cells or Csk^−/−^; Ras^V12^ cells did not result in detectable tumor populations in the other body parts [79]. This strongly suggests that metastasis induced by NEK2A works in conjunction with other pathways, like Ras. Taken together, this data indicates a pivotal role of NEK2A in tumorigenic growth and progression; however the underling mechanisms are still poorly understood.

In a previous study, we showed that the AKT inhibitor LY294002 and *β*-catenin shRNA decrease the NEK2A induced colony formation in multiple myeloma, suggesting that both PP1/AKT and the Wnt signaling pathway may be involved in NEK2A-induced cell proliferation [12]. Evidence of NEK2A involved in Wnt signaling has been uncovered by other groups as well. An excellent example comes from Neal et al. in colorectal cancer [54]. In this study, NEK2A overexpression was associated with lower tumour membranous *β*-catenin expression and higher cytoplasmic and nuclear *β*-catenin accumulation [54]. Our previous study also showed that overexpressions of NEK2A in multiple myeloma and lung cancer cells induce nuclear accumulation of *β*-catenin [12]. *β*-Catenin localization from the intercellular adherens junction to the cytoplasm and nucleus is characteristic of tumor metastasis; thus NEK2A may play an important role in tumor metastasis through regulating the expression and localization of *β*-catenin. Our preliminary data also showed that NEK2A increases *β*-catenin transcriptional activity and exhibits role of antisenescence through increasing phosphorylation of Rb (unpublished data).

### 4.3. Drug Resistance

Drug resistance is one of the main problems in cancer treatment. Our previous studies have implicated NEK2A in cancer cell drug resistance [12]. Multiple myeloma cells transfected to overexpress NEK2A showed only a slight decrease in their capacity to form colonies when treated with Bortezomib, doxorubicin, and Etoposide. However, control cells transfected with empty vectors showed a significant decrease in colony formation when incubated with these drugs at the same concentrations. Studies from another research group showed that both NEK2A and polo-like kinase 4 (PLk4) are highly expressed in Her2-positive breast cancer cells exhibiting trastuzumab resistance [52]. NEK2A expression is upregulated in drug-resistant ovarian cancer cells as well, when compared with their sensitive or parental counterparts. Thus it is clear that NEK2A has a role in cancer cell drug resistance. To understand how NEK2A generates this resistant phenotype, we conducted flow cytometry in search for apoptotic cells. The results indicated that multiple myeloma cells overexpressing NEK2A showed lesser cell apoptosis after treatment with anticancer drugs than control cells without NEK2A overexpression. Consistently, shRNA-mediated NEK2A depletion overcame myeloma cell drug resistance and induced apoptosis in vitro and in a xenograft myeloma mouse model [12]. A bioinformatic analysis consisting of protein/gene-protein/gene interaction networks, annotation of biological processes, and microRNA-mRNA interaction indicated that NEK2A directly or indirectly interacts with a number of genes, proteins, and microRNAs [80]. This study also suggested NEK2A had implications in biological processes associated with drug resistance in ovarian and other types of cancer [80]. In our study, Western blot results showed that overexpression of NEK2A in cancer cells upregulated ABC transporter family members, including ABCB1 (p-glycoprotein, MDR1), the multidrug resistance protein ABCC1 (MRP1), and the breast cancer resistant protein ABCG2 [12]. Consistently, downregulation of NEK2A by shRNA decreased the expression of these ABC transporters. To corroborate that the NEK2A-induced increase of ABC transporters contributes to drug resistance, a flow cytometry-based analysis was performed. This showed that cancer cells overexpressing NEK2A have a higher efflux of the hydrophilic eFluxx-ID gold fluorescent dye compared with control cells, indicating higher activity of ABC transporters in NEK2A-elevated cancer cells. Verapamil, an ABC transporter inhibitor, was able to abrogate part of the NEK2A-induced drug resistance by showing a decrease in colony formation. Our data strongly suggest that NEK2A induces drug resistance mainly through enhancing the activation of ABC transporters. Our subsequent studies further indicated that both PP1/AKT and canonical Wnt signaling were involved in NEK2A-induced activation of ABC transporters [12]. Inhibition of AKT or knockdown of *β*-catenin in NEK2A-overexpressed myeloma cells inhibits the expression of ABC transporters ABCB1, ABCC1, and ABCG2; moreover, there was a decreased efflux of the hydrophilic eFluxx-ID gold fluorescent dye in those cells. This suggests that NEK2A induction of ABC transporters involves AKT and *β*-catenin. In addition, we found that overexpression of NEK2A in cancer cells suppressed the expression of the proapoptotic genes BAD and PUMA and upregulated the expression of prosurvival genes BCL-XL and MCL-1 [12]. Depletion of NEK2A in cancer cells increased the level of cleaved PARP and activation of caspase-3, caspase-8, and caspase-9, indicating a possible role of NEK2A against the apoptosis pathway [12]. The other group also found that NEK2A knockdown in breast cancer cells induces aneuploidy, cell cycle arrest, and caspase-dependent and -independent cell death. Mechanistically, NEK2A depletion in breast cancer cell increases caspase-3 cleavage and promotes the activity of the tumor suppressor Rb while simultaneously reducing the activation of the cell division regulator histone H3 [65]. Because induction of apoptosis is one of the main mechanisms of anticancer drugs use to stimulate cell death, NEK2A-induced antiapoptosis may explain the high cancer cell drug resistance seen when NEK2A is increased.

**Figure 2:**
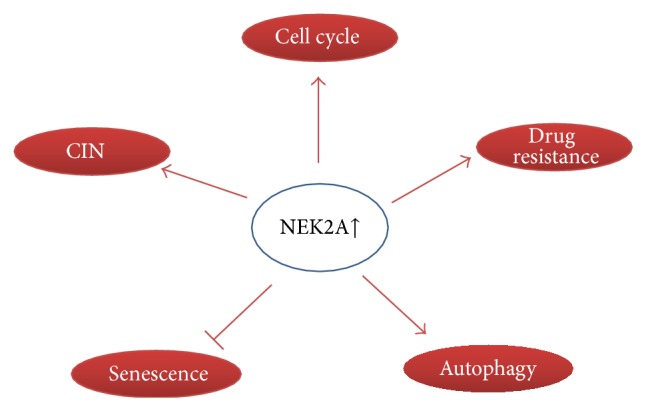
Summary of oncogenic activity of NEK2A.

Many cancers avoid apoptosis and generate drug resistance after chemotherapeutic agents by activating prosurvival mechanisms like autophagy [81]. Many independent groups have shown that autophagy can antagonize apoptosis and other forms of cell death after drug treatment [80]. This is particularly important for multiple myeloma, a cancer high in NEK2A expression and elevated autophagic flux [82]. NEK2A has been shown to alter pathways like AKT and be activated by MAPK, as discussed previously. Because these two pathways are important modulators of autophagy, it is likely that NEK2A could be altering autophagy, as a means to sustain malignant cells after drug treatment. Increased autophagy by NEK2A could be a novel mechanism by which cancer cells acquire drug resistance; however, to our knowledge, no group has yet exploited this approach. The study of autophagy regulation by NEK2A could provide more insight on the currently misunderstood NEK2A-derived malignancy and also the autophagic process. We summarized oncogenic function of NEK2A in Figure 2.

## 5. Therapeutic Potential of NEK2A

The rationale for exploring the therapeutic potential of NEK2A is based on the observations described above that implicate NEK2A in various human cancers, contributing to tumorigenesis, tumour progression, and drug resistance. In recent years, several studies focused on the relationship between NEK2A and cancer clinicopathological factors. To explore the roles of NEK2A in human breast cancer progression, researchers correlated the expression of NEK2A with some of the clinicopathological factors in human breast cancer tissue. As a result, NEK2A mRNA expression was associated with certain molecular subtypes, like Estrogen Receptor (ER), Progesterone Receptor (PR), and Ki-67 immunoreactivity (*P* < 0.05) in breast ductal carcinoma in situ (DCIS) tissue; moreover, in IDC tissue, NEK2A expression was associated with histological grade, lymph node metastasis, molecular subtypes, C-erbB-2 expression, and Ki-67 expression (*P* < 0.05) [49]. Breast cancer patients with high expression of NEK2A exhibited higher mortality and recurrence rate than NEK2A low expression patients. In human pancreatic cancer, overexpression of NEK2A was significantly correlated with histological differentiation (*P* = 0.042), lymph node metastasis (*P* = 0.003), and tumor stage (*P* = 0.001) [58]. Pancreatic cancer patients with a high NEK2A expression also had a significantly worse overall survival than those patients with low NEK2A expression (*P* = 0.002). Likewise, nonsmall cell lung cancer patients with overexpression of NEK2A also had a poorer overall survival rate compared to those with low expression for all stages (*P* = 0.000) [56]. Colorectal cancer patients with high NEK2A expression had a significantly poorer prognosis than those with low NEK2A expression [68]. Moreover, univariate and multivariate analysis showed that NEK2A mRNA expression was an independent prognostic indicator of overall survival in patients with colorectal cancer [68]. In addition, our previous A Kaplan-Meier survival analysis has indicated that high expression of NEK2A is linked to poor survival in multiple myeloma [12]. The same clinical implication of high NEK2A expression is also observed in other cancers, including acute myeloid leukemia, bladder cancer, breast cancer, glioma, lung adenocarcinoma, mantle cell lymphoma, and mesothelioma [12]. Taken together, those data suggest that NEK2A is a novel potential biomarker for diagnosis and a possible therapeutic target for cancer.

Overexpressing NEK2A in cancer cells resulted in enhanced cancer progression and drug resistance, while targeting NEK2A with shRNA overcame cancer cell drug resistance and induced apoptosis. Therefore, downregulation or inactivation of NEK2A in cancer cells may contribute to cancer therapy. In recent years, based on the spatial structure of NEK2A, a number of specific NEK2A inhibitors have been developed through high-throughput screening [83–86]. A small molecular inhibitor for NEK2A and HEC1 binding 1 (INH1) has been first found to specifically disrupt the HEC1/NEK2A interaction via direct HEC1 binding thereby leading to metaphase chromosome misalignment, spindle aberrancy, and eventual cell death [87]. Treatment with INH1 suppresses the proliferation of multiple human breast cancer cells in vitro. In vivo, INH1 retarded tumor growth in a nude mouse model bearing xenografts derived from the human breast cancer line MDA-MB-468, with no apparent side effects. In recent years, researchers successively developed many more effective INH, such as INH41 [88], INH154 [88], TAI-1 [89], and TAI-95 [90]. These inhibitors had IC_50_ in nm level and suppressed the growth of multiple types of cancer cells but had no significant growth inhibitory effects on the nontumorigenic cells [88–90]. In addition, these inhibitors not only disrupt HEC1-NEK2A protein interaction but also promote NEK2A degradation through the proteasome pathway and may act as powerful cancer therapeutic for NEK2A and HEC1 overexpressing cancers. A study examining the effect of a combination treatment using NEK2A siRNA with the chemotherapeutic agent cisplatin (CDDP) on a colorectal cancer model indicated that administration of NEK2A siRNA with CDDP results in the suppression of tumor growth compared to the single administration of NEK2A siRNA or control siRNA and CDDP [53]. Targeting NEK2A by siRNA or antisense oligonucleotides (ASOs) in breast cancer cells increased drug sensitivity. These results suggest that combination treatment using NEK2A siRNA and chemotherapeutic agents may be effective and can serve as a therapeutic option for the treatment of cancer.

## 6. Conclusion

As reviewed above, NEK2A contributes to several biological processes of the tumor cell, including proliferation, metastasis, and drug resistance. Studies from our group and others have indicated that elevated expression of NEK2A is positively correlated with molecular subtypes, tumor stage, poor prognosis, and poor overall survival rate. These studies together suggest that NEK2A may be a novel potential therapeutic target for human cancers. Because NEK2A has such a broad spectrum of roles in different cell processes, it is expected that by targeting this kinase, several tumor promoting pathways will be affected, greatly improving treatment outcome. Some research groups have already developed various NEK2A inhibitors, which have been shown to effectively suppress tumor growth in xenograft nude mouse model; thus the outlook on this field appears promising. On the other hand, because initial data has shown NEK2A can predict patient prognosis, more research on its efficiency in predicting disease stage and overall outcome is greatly encouraged, since this data can help generate a more personalized and efficient treatment for the cancer patient in the future. Overall, we anticipate that further studies will provide more convincing support for NEK2A-based therapy strategies for various cancers.

## Figures and Tables

**Table 1:** NEK2A interaction proteins and their functions.

NEK2A interaction protein	Detection method	Function	Reference number
APC/C	Co-IP	NEK2A degradation	[23]
PP1	Yeast two-hybrid, Co-IP	NEK2A dephosphorylation	[26, 27]
C-Nap1	Yeast two-hybrid	Centrosome separation	[28]
Rootletin	Yeast two-hybrid	Centrosome separation	[29]
NLP2	Yeast two-hybrid	Microtubule organization	[33]
Numatrin	Co-IP, pull-down	Centrosome integrity and dynamics	[37]
HMGA2	Co-IP, pull-down	Chromatin condensation	[38]
HEC1	Co-IP	Spindle assembly checkpoint, chromosome separation	[42]
MAD1	Yeast two-hybrid, Co-IP	Spindle assembly checkpoint, chromosome separation	[40]
TRF1	Yeast two-hybrid, pull-down	Chromosome separation	[41]
MAD2	Co-IP	Spindle assembly checkpoint, chromosome separation	[12]
SGO1	Pull-down, Co-IP	Chromosome congression	[43]

**Table 2:** Aberrant expression of NEK2A in different cancers.

Cancer type	NEK2A upregulation	Detect method	Reference number
Ewing tumor	Cancer cell line	DNA microarray analysis	[45]
Cholangiocarcinoma	Cancer cell line	RT-PCR, Western blot	[46]
Testicular seminomas	Cancer cell line and tumor tissue	Immunohistochemistry, Western blot	[47]
Breast cancer	Cancer cell line and tumor tissue	RT-PCR, Western blot, immunohistochemistry, and DNA microarray analysis	[48–52]
Cervical cancer	Cancer cell line	Western blot	[50]
Prostate cancer	Cancer cell line	Western blot	[50]
Colorectal cancer	Cancer cell line and tumor tissue	Western blot, DNA microarray analysis, and immunohistochemistry	[53, 54]
Malignant peripheral nerve sheath tumors	Tumor tissue	DNA microarray analysis, immunohistochemistry	[55]
Lung adenocarcinoma	Tumor tissue	DNA microarray analysis, immunohistochemistry, immunofluorescence	[12, 56]
Renal cell carcinoma	Tumor tissue	DNA microarray analysis	[57]
Pancreatic ductal adenocarcinoma	Tumor tissue	Real-time PCR, immunohistochemistry	[58]
Multiple myeloma	Cancer cell line and tumor tissue	DNA microarray analysis	[12]
Myeloid leukemia	Tumor tissue	DNA microarray analysis	[12]
Mantle cell lymphoma	Tumor tissue	DNA microarray analysis	[12]
Mesothelioma	Tumor tissue	DNA microarray analysis	[12]
Head and neck squamous cell carcinoma	Tumor tissue	DNA microarray analysis	[12]
Bladder carcinoma	Tumor tissue	DNA microarray analysis	[12]
Glioblastoma	Tumor tissue	DNA microarray analysis	[12]
T cell acute lymphoblastic leukemia	Tumor tissue	DNA microarray analysis	[12]
Hepatocellular carcinoma	Tumor tissue	DNA microarray analysis	[12]
Melanoma	Tumor tissue	DNA microarray analysis	[12]
Ovarian adenocarcinoma	Tumor tissue	DNA microarray analysis	[12]

**Table 3:** Signaling involved in the tumorigenic function of NEK2A.

Proteins or signaling pathways interact with NEK2A	The relationship with NEK2A	Function	Reference number
*Upstream of NEK2A *			
MAPK pathway	Phosphorylate NEK2A	Tumorigenesis	[39]
MST2	Regulate NEK2A's ability to localize to centrosome and phosphorylate C-Nap1 and Rootletin	Tumorigenesis	[59]
PLK1	Regulate MST2-NEK2A/NEK2A-*β*-catenin-induced centrosome separation and NEK2A-NIP-induced microtubule organization	Tumorigenesis	[32, 60, 61]
CDK4	Regulate NEK2A expression	Tumorigenesis	[62]
*Downstream of NEK2A *			
CDC20	Phosphorylated by NEK2A	Tumorigenesis	[63]
MAD2	Phosphorylated by NEK2A	Tumorigenesis	[63]
HEC1	Phosphorylated by NEK2A	Tumorigenesis	[42]
TRF1	Phosphorylated by NEK2A	Tumorigenesis	[64]
AKT	Phosphorylated by NEK2A	Tumor progression and drug resistance	[12]
*β*-Catenin	NEK2A induces nuclear accumulation of *β*-catenin	Tumor progression and drug resistance	[12]
ABCB1	Upregulated by NEK2A	Drug resistance	[12]
ABCC1	Upregulated by NEK2A	Drug resistance	[12]
ABCG2	Upregulated by NEK2A	Drug resistance	[12]
BAD	Downregulated by NEK2A	Drug resistance	[12]
PUMA	Downregulated by NEK2A	Drug resistance	[12]
BCL-XL	Upregulated by NEK2A	Drug resistance	[12]
MCL-1	Upregulated by NEK2A	Drug resistance	[12]
PARP	Activated in NEK2A silenced cancer cell	Drug resistance	[12]
Caspase-3	Activated in NEK2A silenced cancer cell	Drug resistance	[12]
Caspase-8	Activated in NEK2A silenced cancer cell	Drug resistance	[12]
Caspase-9	Activated in NEK2A silenced cancer cell	Drug resistance	[12]
RB	Activated in NEK2A silenced cancer cell	Drug resistance	[65]
Histone H3 (p-Ser10)	Inactivated in NEK2A silenced cancer cell	Drug resistance	[65]

## Abbreviations

CIN: Chromosome instability
PLK1: Polo-like kinase 1
NEK2A: Never in Mitosis (NIMA) Related Kinase 2A
APC/C: Anaphase Promoting Complex
PP1: Protein Phosphatase 1
NLP2: Ninein-like protein 2
SAC: Spindle assembly checkpoint
MAD1: Mitotic arrest deficient-like 1
MAD2: Mitotic arrest deficient-like 2
TRF1: Telomere repeat binding protein 1
HEC1: Highly expressed in cancer 1
ETs: Ewing tumors
GEP: Gene expression profiling
RB: Retinoblastoma
FOXM1: Forkhead transcription factor 1
CA: Centrosome amplification
CDC20: Cell division cycle 20 homolog
IDC: Invasive ductal carcinoma
DCIS: Ductal carcinoma in situ
ER: Estrogen Receptor
PR: Progesterone Receptor
INH1: Inhibitor for NEK2A and HEC1 binding 1.
